# Terrorist bombing

**DOI:** 10.1186/1749-7922-1-33

**Published:** 2006-11-13

**Authors:** Ami Mayo, Yoram Kluger

**Affiliations:** 1 General Surgery B, Rambam Medical Center, Haifa, Israel; 2 Rapaport School of Medicine, Technion, the Israel Institute of Technology, Haifa, Israel

## Abstract

Bombings and explosion incidents directed against innocent civilians are the primary instrument of global terror. In the present review we highlight the major observations and lessons learned from these events. Five mechanisms of blast injury are outlined and the different type of injury that they cause is described. Indeed, the consequences of terror bombings differ from those of non-terrorism trauma in severity and complexity of injury, and constitute a new class of casualties that differ from those of conventional trauma. The clinical implications of terror bombing, in treatment dilemmas in the multidimensional injury, ancillary evaluation and handling of terror bombing mass casualty event are highlighted. All this leads to the conclusion that thorough medical preparedness to cope with this new epidemic is required, and that understanding of detonation and blast dynamics and how they correlate with the injury patterns is pivotal for revision of current mass casualty protocols.

## Background

Bombings and explosions directed against innocent civilians are the primary instrument of global terror, resulting in death, injury, fear and chaos. With the lessening of full-scale military conflicts, terror has become a prominent feature in modern life, as realized by the tripling of the number of serious terror incidents in recent years.

Fuelled by zealotry and supported by tools of modern life, especially the Internet, the destructive capabilities of terrorists have increased tremendously. Conventional explosives are the commonest tools of terrorist attacks, because of the easiness by which explosives and knowledge of their manufacture and use can be acquired and the stricter control of stockpiles of nuclear, biological and chemical weapons. In contradiction to the common perception that unconventional weapons are more dangerous than explosives, the number of lives lost and people injured and infrastructure damaged from bombings are orders of magnitude higher than those caused by chemical or biologic incidents. This necessitates fresh awareness, properly oriented education of medical professionals, and careful and advanced planning of the medical system.

In a group of reports we have described various medical aspects of terrorism related trauma, painfully learned from a series of suicide bombings in Israel [1-8]. In the present review we highlight the major observations and lessons learned from these events.

## Mechanisms of blast injury

The general types of common explosives used in terror bombing have been discussed elsewhere [8,9], but all explosives produce their detrimental effects through one or more of the following distinct mechanisms of blast injury, each of which is responsible for a different type of injury.

### Primary blast injury mechanism

The initial blast wave, an almost instantaneous rise of air pressure, is followed by a wave of negative pressure when air gushes to fill the void caused by the primary pressure wave and follows complex nonlinear physics.

The forces of the blast waves decrease exponentially with distance from source and so is their damage. When the same explosive is detonated in confined closed quarters, a distinctive increase in the morbidity and in mortality, compared to open-air explosion, is evident [7,10,11]. In open-air explosions the quick dissipation and velocity decline of the shock front results in low immediate and late-mortality and in predominantly non-critical injuries, but indoors standing waves and heightened pressure differentials from reflection of the waves from walls and objects produce a special pattern of blast behavior, particularly in ultra-confined spaces (eg buses). The localized area of overpressure from the explosion is instantly amplified, and therefore bus bombings are highly lethal [1]. Thus, proximity of the victims to the explosion site is responsible only for part of the high incidence of death, while intense overpressure also further away from the blast center is also the immediate cause of many other deaths.

The blast wave itself causes the primary and tertiary injury patterns; it affects mostly air-filled organs and air-liquid interfaces. Perforation of the eardrums and pneumothoraces are the hallmarks of blast wave injury. Solid and fluid-filled organs are rarely damaged, but their interaction with the blast wave may accelerate and so increase the injurious stress forces.

### Secondary blast mechanism

The wounding potential of the bomb is often amplified by the addition of metallic particles such as nuts, bolts or balls to the charge. They and the fragments of the casing cause penetrating injuries. These are the leading cause of death and injury in terror bombings.

### Tertiary blast mode of action

The wind that follows the wave of high pressure is responsible for the tertiary blast injuries when the victim's body thrusts upon fixed objects [12]. All body parts may be affected, and fractures, traumatic amputations, and open and closed brain injuries occur. Furthermore, structural collapse of buildings, street fixtures and cars that is caused by the wind produces blunt trauma, crush injuries, and entrapment.

### Quaternary blast action

These include burns (chemical or thermal) and exposure to radiation. While conventional explosives generally do not cause primary fires because most of the available oxygen is exhausted during the explosion, these are sometimes encountered. Incendiary bombs sometimes contain powdered aluminium soap or similar compounds that gelatinize or thicken the oil and fuel, and so increase adherence and burning time.

### Quinary blast mechanism

We have noticed a unique early hyperinflammatory state that did not correlate with the complexity of the sustained injury among patients involved in a terrorist attack in Tel Aviv [3]. Toxic effects caused by chemicals that were part of or were especially added to the charge manifest the fifth mechanism of blast injury.

## Patterns of injury

Victims of bombings may present with a tremendously varied pattern of injury. In addition to the unique class of blast injuries, these patients may exhibit the classic manifestations of blunt trauma, as well as penetrating injuries and burns [13]. These injuries are caused by the different mechanisms described above, alone or in tandem, as illustrated below.

### Primary blast injuries

The effect of the blast waves on air containing organs is the most profound. The middle ear, lungs, and the digestive tract are the organs most susceptible to blast injury, with the frequency of presentation in this order.

The eardrum can be injured by a rather small pressure differential of 5 psi [14], but the common belief that eardrum injury is a predictor of other blast injuries was negated by the lack of correlation between other injuries and eardrum perforation studied in a large case series of civilian terror bombing victims, where isolated eardrum perforation in survivors of explosions was not a reliable marker of concealed pulmonary blast injury or of poor prognosis [15].

The lung is the organ second most susceptible to primary blast injury. Pressure differentials across the alveolar-capillary interface cause disruption, haemorrhage, pulmonary contusion, pneumothorax, hemothorax, pneumomediastinum, and subcutaneous emphysema [16]. The complex mechanics of this type of injury, and its biochemical and pathological consequences were described elsewhere [8,17,18]. One lesson learned was the life threatening potential of diffuse interstitial haemorrhage around larger and smaller vessels within the lung parenchyma, which might require thoracotomy immediately upon admission for haemorrhage control [19].

Blast lung should be suspected in any casualty with dyspnea, cough, hemoptysis or chest pain following blast exposure [20]. The management, in a nutshell, is similar to caring for pulmonary contusion, with judicious fluid administration ensuring and supplemental high flow oxygen is sufficient to prevent hypoxemia (for more see [8]) in most victims.

Blast pulmonary injuries are life threatening, and pulmonary barotrauma is the most common fatal primary blast injury. It is also the most common critical injury in people close to the blast center. Early deterioration to acute respiratory distress syndrome (ARDS) has been described [21], and, immediate cardiovascular derangement, resulting in death even in the absence of any demonstrable physical injury may result.

Air embolism is a well-recognized consequence of blast lung injury, and can lead to cardiac dysfunction and immediate death [22]. It is a matter of controversy whether air embolism is the result of mechanical ventilation, but in an autopsy-based study of immediate blast casualties, evidence for air embolism was found in nearly 50%, even though none had been mechanically ventilated [18].

Gastrointestinal (GI) tract blast injuries are rare, yet up to 1.2% of patients exposed to bomb explosions suffer GI blast injury [23], most of them in gas-containing sections of the GI tract, primarily the colon and to lesser extent the small intestine. Perforation of these hollow abdominal viscera depends on the amount of the charge, proximity to the explosion, and the spatial surroundings of the explosion site. Frank bowel perforation may be delayed for hours. Slow mucin dissection between the walls layers of the bowel could be the underlying mechanism [3]. Mesentery tears or mesenteric avulsion leading to bowel wall ischemia and eventual perforation is a more reasonable explanatory mechanism. [4]. This injury should be suspected in anyone exposed to an explosion with findings suggestive of acute abdomen [8]. Ultrasonography and computed tomography may not be of diagnostic assistance at early stages in cases with late presentation of this injury. Diagnostic peritoneal lavage (DPL) might be more useful and accurate. In mass casualty incidents, were the imaging modalities are graded and not initially fully available and the ability for intense follow-up of these patients is difficult this diagnostic tool should be considered. The management of bowel wall contusion management is controversial, and the question of bowel resection versus conservative management remains open [24]. Solid surgical judgment and experience should direct the surgeon.

Other organs suffer true blast injuries as well. Head injuries, responsible for some of the dead-on-scene events, must be considered and their extended lucid interval taking into consideration when deciding on patient admission or transfer.

### Secondary blast injuries

Projectiles like steel balls, nails, screws and nuts packed around the explosive cause secondary blast injuries, and the wounds reflect their velocity and shape. Multiple penetrations of such pellets result in increased mortality and devastating injuries, and such were encountered in many suicide bombing incidents. They were analyzed in detail in one particular bombing event where a suicide bomber detonated an 8 to 10 kg charge packed with hundreds of hard steel balls in the centre of a crowded dining hall of a hotel during the ceremonial Passover dinner [5].

Of the 91 victims with bodily injuries, 20 died on the scene, and among the survivors, all the 32 severely injured (Injury Severity Score (ISS >16) suffered tissue penetration by the spherical pellets. In three patients classical blast injury combined with wounding by the pellets was noted. All 8 pneumothoraces were the result of lung penetrations by spherical pellets combined with blast injury. In the other patients the destructive nature of the organ injuries was limited, and superficial penetrations were easily treatable. Forensic studies on the deceased allowed establishing the organ distribution of body penetration by the missiles; and on average each immediate fatality absorbed 16.6 ± 8.8 pellets (range 3–37). Eighteen victims (90%) had sustained cerebral and facial injuries from the steel balls, and one victim suffered major limb amputation [5].

Multiple penetrations to the human body demand special awareness during evaluation, and all means of evaluation should be used to exclude cardiac or vascular injury in such patients. Liberal use of total body fluoroscopy to identify all potential projectiles and their mapping is mandatory for documentation and future reference. When multiple shrapnel pieces are identified on X-ray or fluoroscopy, increased suspicion is warranted [2].

### Tertiary blast injury

Tertiary blast injury is caused when the blast wind thrusts the victim against stationary objects and by wind disruption. In some patients, the dynamic pressure from the blast wind may result in limb amputation. Major limb amputation serves as a predictor for the severity of injury with many victims fatally overwhelmed in the field. Still, our experience indicated that most of those victims brought in alive, survive and eventually are discharged [4].

Among the effects of this mechanism are shearing forces applied to the lung parenchyma when it decelerates against the chest wall, crush syndrome in victims of structural collapse, and compartment syndrome [4,8]. Deceleration forces caused also injuries to solid organs such as the liver, spleen and kidney. Although some have considered these to be the consequence of the primary blast mechanism causing acceleration-deceleration it is more likely that acceleration and deceleration of solid organs result from the bumping of the body against other objects, resembling classical injury of blunt trauma.

### Quaternary blast injury

The quaternary blast injury is related to the thermal effects of the blast. Burns are caused when flammable materials on the scene are ignited. Thermal lung injury can develop directly from the very high air temperatures at the site of explosion. After incendiary bomb attacks, the number of burn injuries should be ascertained as early as possible and alternative national burn management resources should be alerted because a large number of burn victims can quickly overwhelm local medical resources.

### Quinary blast injury

In one suicide bombing in a nightclub in the city of Tel-Aviv, a hyperinflammatory state was noticed, which did not correlate with the complexity of the sustained injury. It was absent in those injured away from the centre of explosion or among patients who had no skin injury [3]. From this event we have reported on four cases that could indicate the presence of toxic substances absorbed by the casualties through their injuries or via inhalation. It was postulated that the bizarre hyperinflammatory state resulted from absorption of a particular explosive, penta- erythritol- tetra- nitrate (PETN), which possesses vasodilatory properties. However, such hyperinflammation must be differentiated from missed injuries that may express with hemodynamic instability.

## Consequences of terror bombings

### Severity of injury

The consequences of terrorist bombings during a 33 months period were analyzed and compared to those of non-terror trauma, in a retrospective cohort study of the Israeli Trauma Registry records [6,7]. Not only were bombing casualties younger than other trauma patients with half of them in their 20's, but terror bombing also resulted in a significantly different severity and complexity of injury. The severely injured (ISS≥16) were almost trice as frequent, low Glasgow Coma scores (≤5) four-fold more abundant, and hemodynamically unstable arrivals double than in non-terror trauma events. The number of body regions injured, was significantly increased in terror victims, with approximately 18% having 3 injured body regions and 11% four or more regions, compared to 5% and 1.5%, respectively in the non-terror trauma group. Not surprisingly, intensive care admissions and surgical interventions were markedly increased in terror bombings, hospital stay was much extended in all ISS levels, and the immediate and late mortalities were also significantly higher. We have concluded that terrorist bombing has created a new class of casualties that differ from those of conventional trauma. Not only are there differences in the formal severity of injury upon admission but also in the number of body regions injured. Indeed, many bombing victims had more than three regions with most severe injuries, revealing the inadequateness of the ISS for evaluating these patients. The ISS failed also to reflect the severity of outcome in these bombing patients [7].

The different clinical behavior of bombing victims cannot be attributed only on the increased proportion of severely injured patients, since all variables, except intensive care, were also significantly increased in less severely injured victims. Therefore, a Multidimensional Injury Pattern (MIP) has been distinguished [1,4].

### Clinical implications

The MIP is a tremendous challenge to the trauma surgeon, particularly when limited diagnostic information is available. The multiple injury complexes require careful coordination and imply strict supervision of the trauma surgeon among the treating teams that are involved. Prioritization of treatment regimens and liberal use of damage control strategies, while postponing definitive treatment until stabilization is achieved is also required [7]. Treatment dilemmas in the multidimensional injury can be exemplified by the different approaches required for blast lung versus lacerated lung. While lacerated lung may result from secondary blast injury from penetrating missiles, blast lung injury is classical for the first blast injury pattern. Significant respiratory difficulties with persistent air leakage could develop in both, and it requires creative ventilatory practices. Fluid management may be quite different in these two lung injuries in the early phase of injury, because lacerated lung mandates resuscitative therapy while blast lung requires restrictive management, and therefore, ideal tailoring of fluid management and monitoring may be needed. Lacerated lung may occasionally require operative therapy, while the mainstay of blast lung therapy is non-operative respiratory support [4].

### Ancillary evaluation of bombing mass casualty incident

Immediately following a terror bombing incident, not all necessary imaging modalities can be made available to all patients at the same time. Computerized tomography (CT) is initially restricted to head injuries, and only later for identification of missile penetrations and trajectories in other body regions injured (in stable patients).

The presence of metal objects is best depicted by total body fluoroscopy, and this is especially important in unconscious, intubated and ventilated patients. Abdominal x-ray may be indicated in victims as an adjunct to the standard trauma films. Although visualization of a metal object in single-plane film is often inadequate for thorough evaluation, it can direct the team on the need for urgent surgery or for additional imaging.

Because the use of CT is restricted, ultrasonography (US) becomes the primary tool for assessment of the abdomen. Focused assessment with sonography for trauma (FAST) is highly recommended for every patient triaged to a severe or intermediate casualty station [2]. In patients suffering other injuries indicative of blast exposure but with negative US, repeat US assessment should be considered if CT remains unavailable for long period of time. We recommenced liberal use of diagnostic peritoneal lavage in intubated patients and in patients who are rushed to the operating theaters for non-abdominal emergency surgery.

## The medical handling of terror bombing events

Terror bombings mandate hospital preparedness for limited or full-scale multiple casualty incidents with pre-established protocols that can be activated promptly. The location of the terrorist bombing bears upon the hospital's preparation and protocol activation. A remote site allows the admitting hospitals more time to prepare and collect relevant data before the arrival of the first patient. However most suicide bombings have occurred in urban areas near medical centers, and there, sometimes, the arrival of the first victim even precedes the alert. The emergency department and institutional experience gained in the Tel-Aviv Medical Center in handling a deluge of terrorist bombings has been detailed [2]. In post-event debriefing sessions, many topics were identified as critical for proper event handling, and were categorized into functions of key personnel and into definitions and rules. The first category includes the Triage Officer, the Medical Director, the Administrative Director, the Head Nurse, the Emergency Medical System Coordinator, the Blood Bank Liaison, and the Trauma Teams. In the second group, the concept of Triage Hospital, the Unidirectional Patient Flow, ancillary evaluation, the Consultancy, and Tertiary Survey were included. Detailed discussion of these topics was published [2], and below are just a few highlights. Immediately after the explosion, the chaos phase starts and family members, bystanders and passing vehicles evacuate 6%–10% of the injured to the nearest hospital. When trained medical personnel arrive at the scene, the evacuation of the most severely injured patients to the nearest hospital can overwhelm it because at this point it is crowded with patients evacuated in the chaos phase. Over-triage compounds this situation and could lead to the death of patients reaching the hospital alive and otherwise deemed salvageable [13]. Thus, triage protocols for multiple casualty incidents differ from those of other trauma situations [25-27] and all local and regional hospital facilities are recruited to handle the large volume of injured patients.

In the emergency department, the Triage Officer is the first medical professional caring for the victim in the hospital, with the objective to sort patients according to their severity of injury. We have learned that a well-trained and knowledgeable surgical resident can handle this critical task properly until senior staff arrives. Important information regarding the nature of the event, the exact mechanism (explosion in confined or open space), and most importantly, whether all injured have been evacuated should be communicated via the emergency medical services coordinator. When the understaffed emergency department is overloaded, new patients should be diverted to other facilities.

Trauma-oriented teams are assembled as trained trauma staff becomes available, and they attempt to create a microcosm in which Advanced Trauma Life-support (ATLS) guidelines can be followed. The minimum accepted treatment for each patient is ATLS standards. Our protocols provide for the team to remain with the patient from initial evaluation throughout imaging, surgery, if indicated, and transfer to the final destination, to minimize information loss. A senior surgeon should supervise the care of the lightly injured, to recognize under-triage and late manifestations of blast injury.

If manpower is insufficient to follow ATLS guidelines, or if operating theaters or other resources are deficient, the Medical Director should declare the medical center a Triage Hospital. Then, only life-threatening injuries are treated and all other patients are transferred to nearby hospitals after initial evaluation [28].

Implementation of the Trauma Team concept has allowed establishing and adhering to the principle of unidirectional patient flow. This is implemented upon declaration of a multiple casualty incident, as the emergency department is immediately emptied of its patients, and thereafter all bombing victims that are seen and consequently transferred from the ED and do not return to it.

When the acute emergency phase ends, it is crucial to conduct a tertiary survey of all admitted patients. The tertiary survey team differs from the admitting team, usually consisting of an attending surgeon with trauma experience, an orthopedic surgeon, a plastic surgeon, a nurse and a psychiatrist. While mostly only minor injuries were discovered during the tertiary survey, in one event two patients had vascular injuries that were recognized in the tertiary survey when already in intensive care for other severe injuries.

## Conclusion

World terror represents a true modern epidemic that threatens the very survival of the free world. A thorough understanding of detonation and blast dynamics by the treating teams is required to better correlate the injury patterns presented. This is also critical for revision of current multiple casualty protocols. It is up to the medical establishment to prepare suitable protocols, coordinate manpower and secure medical resources to successfully handle terror-bombing events.

## Competing interests

The author(s) declare that they have no competing interests.

## Authors' contributions

AM and YK reviewed the clinical aspects, and YK also reviewed the analysis of trauma cases and the organizational handling of bombing events. All authors read and approved the final manuscript.
